# Genetic association of the gut microbiota with epigenetic clocks mediated by inflammatory cytokines: a Mendelian randomization analysis

**DOI:** 10.3389/fimmu.2024.1339722

**Published:** 2024-06-06

**Authors:** Siyu Tian, Xingyu Liao, Siqi Chen, Yu Wu, Min Chen

**Affiliations:** ^1^ School of Clinical Medicine, Chengdu University of Traditional Chinese Medicine (TCM), Chengdu, China; ^2^ Department of Colorectal Surgery, Hospital of Chengdu University of Traditional Chinese Medicine, Chengdu, China

**Keywords:** gut microbiota, epigenetic clocks, inflammatory cytokines, Mendelian randomization analysis, mediation

## Abstract

**Background:**

A new aging biomarker epigenetic clock has been developed. There exists a close link between aging and gut microbiota, which may be mediated by inflammatory cytokines. However, the relationship between the epigenetic clock, gut microbiota, and the mediating substances is unclear.

**Methods:**

Two large genome-wide association meta-analyses were analyzed by two-sample Mendelian randomization. The results between gut microbiota and epigenetic clock were investigated using the four methods (Inverse variance weighted, MR-Egger, weighted median, MR-PRESSO). Genetic correlation was measured by Linked disequilibrium score regression (LDSC). The correctness of the study direction was checked by the Steiger test. Cochran’s Q statistic and MR-Egger intercept were used as sensitivity analyses of the study. The two-step method was used to examine the mediating role of inflammatory cytokines. We use the Benjamini-Hochberg correction method to correct the P value.

**Results:**

After FDR correction, multiple bacterial genera were significantly or suggestively associated with four epigenetic clocks (GrimAge, HannumAge, IEAA, PhenoAge). And we detected several inflammatory factors acting as mediators of gut microbiota and epigenetic clocks.

**Conclusion:**

This study provides genetic evidence for a positive and negative link between gut microbiota and aging risk. We hope that by elucidating the genetic relationship and potential mechanisms between aging and gut microbiota, we will provide new avenues for continuing aging-related research and treatment.

## Introduction

Aging has been an area of particular concern to humans throughout history. One of the characteristics of aging is epigenetic aging (1). Because most clinical biomarkers are inadequate to represent the underlying mechanisms of aging, it has been difficult to identify molecular targets for interventions for human health longevity (2). Recently, research has shown that the “epigenetic clock”, which is a biomarker of aging found at specific cytosine-phospho-guanine (CpG) sites, can provide accurate age estimates for any tissue or organ throughout the human life course (3). The emergence of epigenetic clocks may help solve many long-standing questions, such as the central question of aging, “How do we get old?”.

The epigenetic clock acts as a heritable indicator of the DNA of biological aging by capturing the unique characteristics of epigenetic aging based on different CpG sites (4). HannumAge (5) and Horvath (6) clocks constitute the inaugural generation of epigenetic clocks, predicting chronological age utilizing DNA methylation data. These methodologies have been extensively applied across blood samples and 51 distinct human tissue and cell types. HannumAge delineated 71 age-associated CpG sites within blood samples (6), whereas HorvathAge ascertained 353 age-related CpG sites across various human tissues and cell types, with adjustments made for blood cell counts (6). Intrinsic Epigenetic Age Acceleration (IEAA) as a derivative of Horvath was developed after the removal of blood cell composition estimates (7). A second representative epigenetic clock, PhenoAge (Levine et al., 2018) and GrimAge (7), predicts associated morbidity and mortality by combining some information about risk and age (e.g. smoking, plasma protein levels, white blood cell counts). PhenoAge included data on 9 clinical biomarkers associated with mortality and 513 CpGs (8). GrimAge included data on seven plasma proteins and 1030 CpGs associated with smoking (7). The second generation of representative genetic clocks can measure the incidence of various diseases and is better at predicting mortality than the first generation (8, 9). GrimAge outperforms PhenoAge and first-generation epigenetic clocks in predicting the time of death (10, 11).

At present, multiple studies has proved that gut microbiota occupies an important position in the aging process (12–14). Dysregulation of gut microbiota is implicated in the modulation of immune and inflammatory responses during the aging process and is associated with the onset of numerous age-related diseases, both intestinal and systemic (13). Interestingly, from the perspective of interactions between gut microbes, inflammatory mediators, and the immune system, the regulation of gut microbiota may help promote both physiological and non-pathological aging processes and may be a potential target for aging interventions (12). However, the genetic relationship and mechanisms of gut microbiota and aging are unclear, and no researchers have explored the causal relationship between gut microbiota and aging from the perspective of epigenetic clocks. Therefore, we use Mendelian randomization (MR) as a novel method that can be used to study genetic associations and causality between the gut microbiota and the epigenetic clock.

MR is a statistical method to assess the causal relationship between the genetic variation associated with exposure and the outcome (15). Compared with traditional observation methods, MR is less affected by residual confounding and reverse causation (16). In the MR Analysis, we are not only interested in the link between epigenetic clocks and gut microbiota but also in the mechanism of how exposure affects the outcome. Mediation MR Analyses can attempt to determine the causal pathways by which exposure affects outcomes and their relative importance. Mediating MR Analysis can identify factors mediating between exposure and outcome, and interventions on these mediating factors can mitigate or enhance the impact of exposure on outcome (17).

Consequently, we conducted a two-sample MR Analysis to investigate the association between gut microbiota and the epigenetic clock. Additionally, a mediation MR Analysis was employed to elucidate the mechanistic role of inflammatory cytokines in the relationship between gut microbiota and the epigenetic clock.

## Methods

### Research description

**Figure 1:**
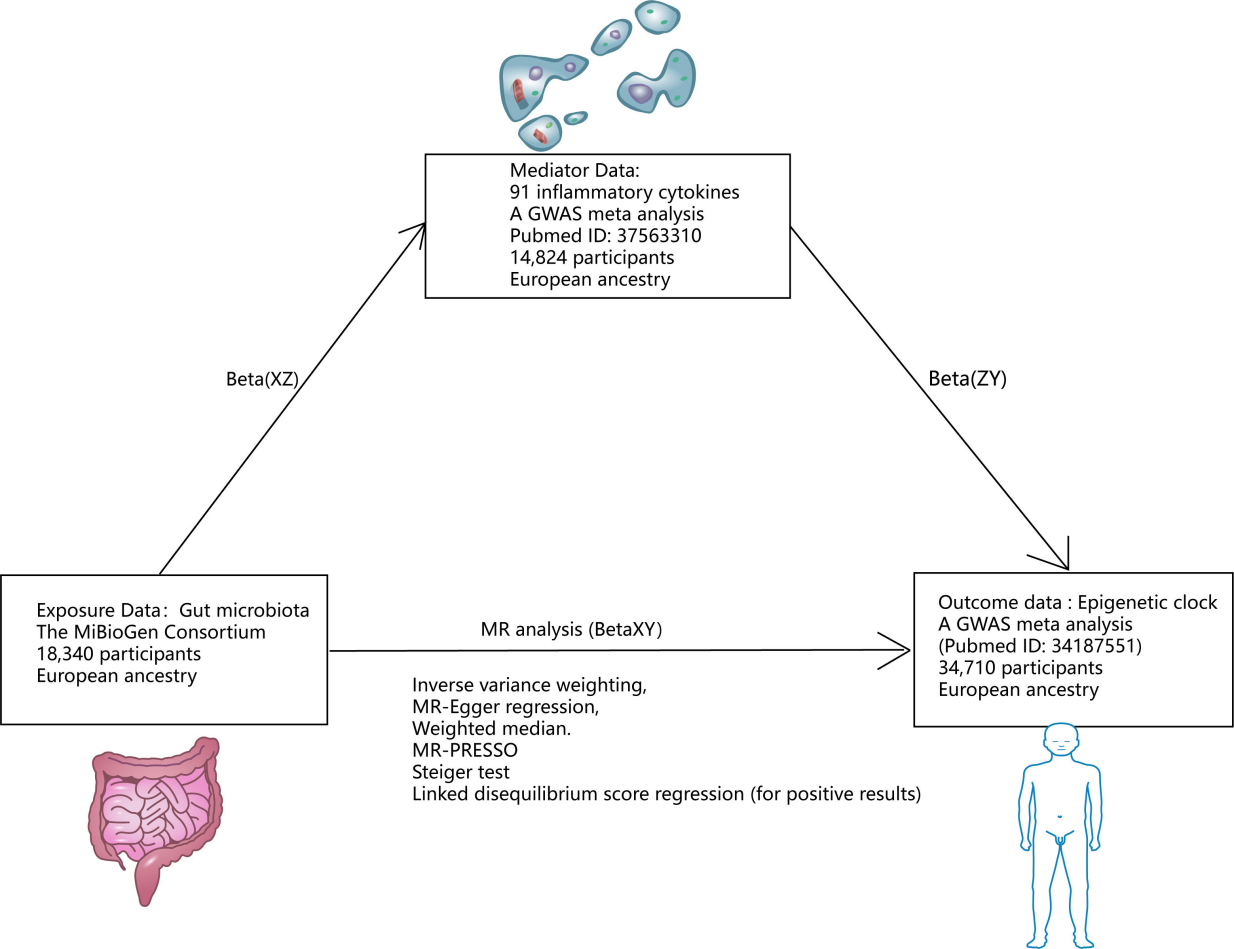
Research design. *Mediating effect =Beta(XZ) x Beta(ZY); Direct effect =Beta(XY)-Beta(XZ) x Beta(ZY).

**Figure 2:**
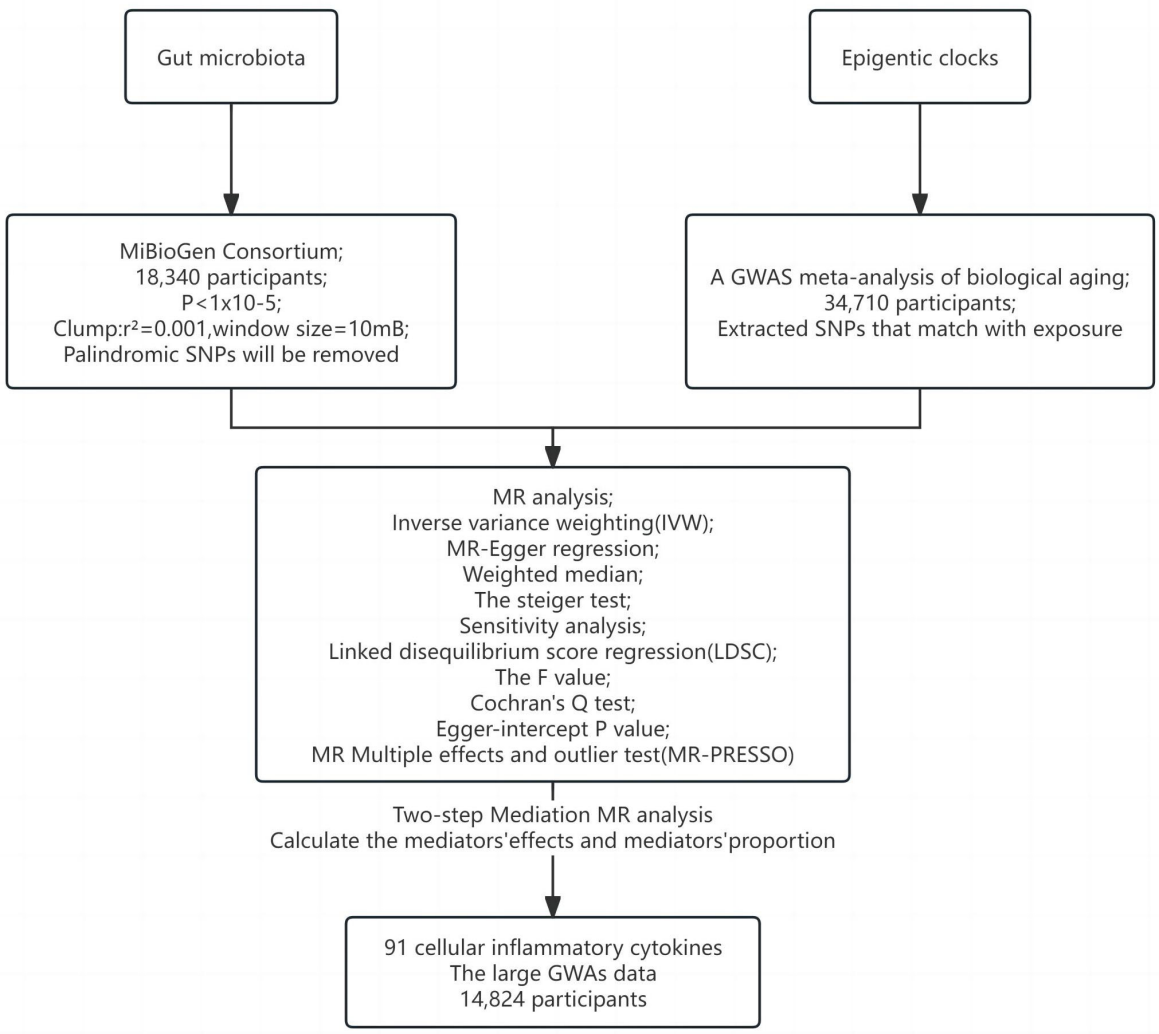
Study flow chart.

**Figures 1**, **2** illustrate the MR research description. Two-sample Mendelian randomization analysis was performed to analyze the link between gut microbiota and the epigenetic clock. Instrumental variables independent of confounding factors such as sex and age were used in the MR Analysis to simulate the random assignment of progeny single nucleotide polymorphisms (SNPs) in randomized controlled trials (RCTS). In addition, the MR design must satisfy three assumptions: (i) genetic tools are correlated with exposure; (ii) genetic tools are independent of potential confounding factors; (iii) Genetic instrumental variables affect results only through exposure. We then used a two-step method mediated MR Analysis to analyze the mediating role of inflammatory cytokines between gut microbiota and the epigenetic clock.

### Exposure data source

Gut microbiota genetic variation data comes from the MiBioGen Consortium (https://mibiogen.gcc.rug.nl/), which is by far the largest gut microbiota genome-wide meta-analysis (18). 18340 individuals were included to analyze the composition of microorganisms in the variable regions of 16S rRNA genes V4, V3-V4, and V1-V2. By mapping microbiota quantitative trait loci (mbQTL), the relationship between host genetic variation and bacterial species abundance in gut microbiota was identified. 131 genera with an average abundance greater than 1% were identified (of which 12 were unknown). Therefore, 119 genera were included in this study for MR Analysis. The instrumental variables (IVs) of gut microbiota were chosen as follows (1): Significant SNPs at the genome-wide level (P < 1×10–5) (19); (2) SNP aggregation using PLINK algorithm (r^2 = ^0.001, window size = 10mB); (3) Palindromic SNPs will be removed (20).

### Outcome data source

Genetic associations of epigenetic clocks (HannumAge, IEAA, PhenoAge, and GrimAge) in 34,710 European participants were derived from a recent GWAS meta-analysis of biological aging (21). Of the 28 subjects of European descent in the study, women participated in 57.3% of the studies. Horvath epigenetic age calculator software (https://dnamage.genetics.ucla.edu) was used in the study or independent script age-adjusted estimate of DNA methylation HannumAge, IEAA, PhenoAge, GrimAge. Abnormal samples of clock methylation estimates that differ by +/-5 standard deviations from the mean will be excluded. Quality control and interpolation procedures were systematically applied across each study. For each cohort, the GWAS summary statistics underwent refinement through adjustments for sex and genetic principal components employing an additive linear model. Then, the data of different races were analyzed by METAL software using the inverse variance fixed-effect scheme (22). Summary statistics were processed and coordinated for each cohort study using the R software package EasyQC (23).

### Mediator data source

Data on the genetic variation of 91 cellular inflammatory cytokines were obtained from the latest large GWAS data, published in August 2023 (24). The investigation quantified 91 inflammatory cytokines across 14,824 subjects and conducted a genome-wide protein quantitative Trait Locus (pQTL) analysis utilizing the Olink Target platform. This was subsequently followed by a meta-analysis of the collected data. These data were combined with disease GWASs to represent the impact of disease-associated variants. MR And mediation analyses are used to identify proteins that are causally linked to the cause of immune-mediated disease.

### Statistical analysis

First, a two-sample MR Analysis was performed for 4 epigenetic clocks and gut microbiota. The random effects inverse variance weighting (IVW) was used as the main analysis result. The F-value was used to measure the potency of instrumental variables (IVs) to test whether this study might violate the first MR Hypothesis (25). Cochran’s Q test was used to quantify the heterogeneity of IVs (26). Horizontal pleiotropy may violate the third MR Hypothesis. We used the MR-Egger regression (27), weighted median (28) method, and MR Multiple effects and outlier test (MR-PRESSO) (29) to test and attempt to correct possible violations of the second and third MR Assumptions. In the weighted regression model, MR-Egger realizes directional pleiotropy by intercept. A value where the intercept term significantly deviates from zero suggests the existence of horizontal pleiotropy (27). The weighted median method sorts the MR Estimates obtained using each IV and then weights the reciprocal of its variance. Individual MR Estimates are provided by median results (27). The weighted median assumes that at least half of the tools are valid and do not require any pleiotropy to affect the intermediate phenotype (30). The SNP results from MR-PRESSO exposure were regressed and the square of the residual was used to identify outlier SNPS that may have pleiotropic effects (29). At the same time, we consider the reverse causality between the gut microbiota and the epigenetic clock, so we use the Steiger test to ensure that our directionality is accurate and that P < 0.05 is significant (31). We employed linkage disequilibrium score regression (LDSC) (available at https://github.com/bulik/ldsc) to evaluate the genetic correlation between Mendelian Randomization (MR) positive outcomes for gut microbiota and epigenetic clocks (32). LDSC represents a robust methodology for the analysis of genetic correlations across complex diseases or traits. It is capable of differentiating between genuine polygenic signals and confounding biases, such as population stratification, among others. If the genetic association is statistically significant as well as by LDSC analysis, we can be sure of a causal association between the two genetic phenotypes (33). When negative genetic particles are present in the sample, the LDSC will not be able to produce results (34). Because LDSC only considers genetic correlations, causation cannot be judged (35). Therefore, when the results of LDSC are inconsistent with the analysis result of MR Analysis, we focus on the analysis result of MR Analysis.

In order to explore the mechanism of positive gut microbiota and epigenetic clock outcomes, we used two-step mediated MR To explore the mediated association of 91 inflammatory cytokines between positive gut microbiota and epigenetic clock. We then screened for mediating inflammatory cytokines associated with positive gut microbiota and epigenetic clocks based on the following criteria (1): There is a genetic association between the epigenetic clock and gut microbiota. (2) There is a genetic association between the mediating inflammatory cytokines and gut microbiota, and the effect of education on mediating should be one-way, because if there is a bidirectional relationship between the two, the effectiveness of mediation analysis may be affected (36). (3) There is a genetic association between the epigenetic clock and inflammatory cytokines and the epigenetic clock. The detailed selection of mediators, as well as the calculation of mediators’ effect and mediators’ proportion are shown in **Figure 1**.

R (version 4.3.1), TwoSampleMR (0.5.5), Mendelian Randomization (0.5.0), MR-PRESSO, and LDSC software packages (37–39) were used for all analyses. The False Discovery Rate (FDR) method was used to correct P values, according to the Benjamin and Hochberg (BH) method. When q < 0.1, the results were significant. While P < 0.05 but q > 0.1 was considered suggestive of causality.

## Results

**Figure 3:**
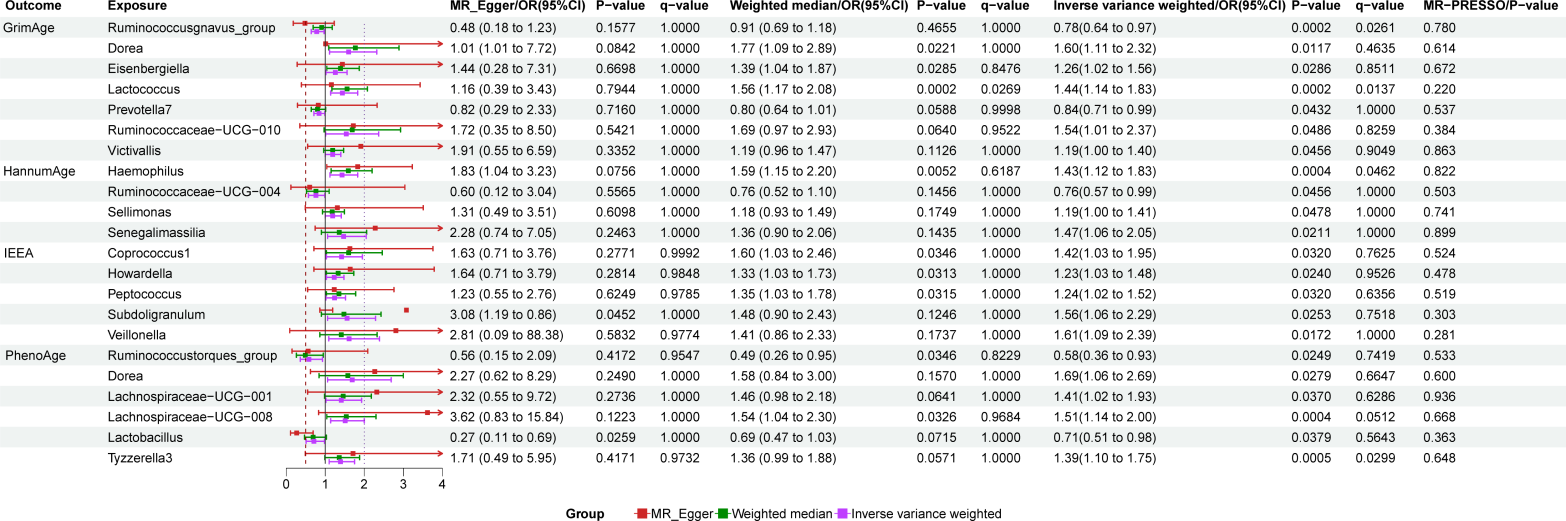
Forest map of gut microbiota and epigenetic clock positive results.

**Figure 4:**
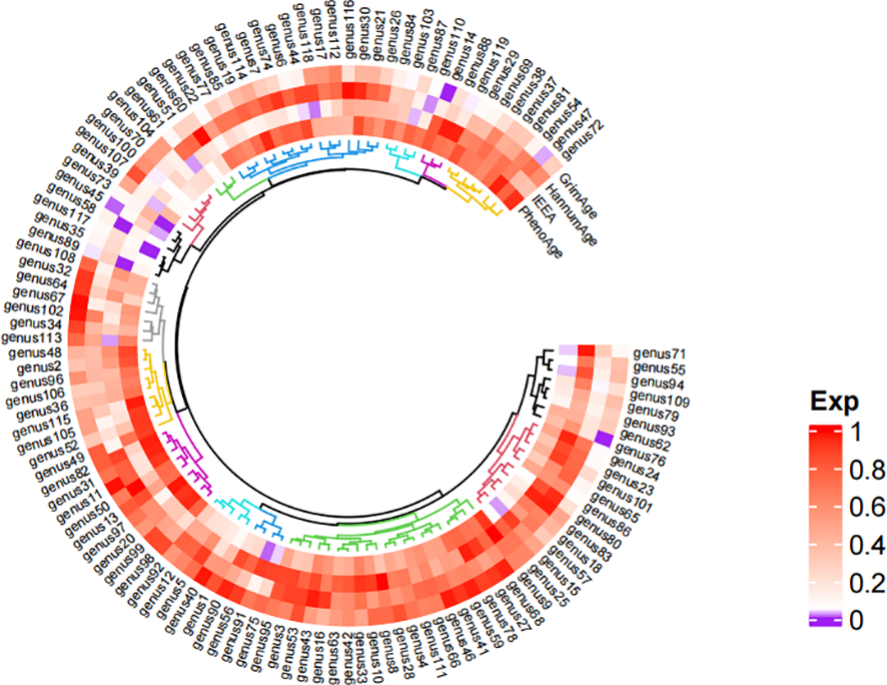
Heat map of the results of Mendelian randomized analysis of gut microbiota and epigenetic clock. *Purple represents positive results, and white and red represent negative results. The comparison table of gut microbiota is in the **Supplementary Material**.

Detailed information regarding the selected instrumental variables (IVs) is presented in **Supplementary Table S1** of the **Supplementary Material**. The F-statistic for each IV exceeds 10, signifying the absence of weak instrumental variables within this study. The positive MR Results are shown in **Tables 1**, **2** and **Figure 3**. All MR Results are shown in **Figure 4** and **Supplementary Materials**.

**Table 1:** Genetic association of gut microbiota and epigenetic clock.

Outcome	Exposure	Methods	P-value	OR(95%CI)	q-value	LDSC rg_p
GrimAge	*Ruminococcusgnavus_group*	MR Egger	0.1577	0.48 (0.18–1.23)	1.0000	0.04
		Weighted median	0.4655	0.91 (0.69–1.18)	1.0000	
		Inverse variance weighted	0.0002	0.78 (0.64–0.97)	0.0261	
GrimAge	*Dorea*	MR Egger	0.0842	2.79 (1.01–7.72)	1.0000	
		Weighted median	0.0221	1.77 (1.09–2.89)	1.0000	
		Inverse variance weighted	0.0117	1.60 (1.11–2.32)	0.4635	
GrimAge	*Eisenbergiella*	MR Egger	0.6698	1.44 (0.28–7.31)	1.0000	0.28
		Weighted median	0.0285	1.39 (1.04–1.87)	0.8476	
		Inverse variance weighted	0.0286	1.26 (1.02–1.56)	0.8511	
GrimAge	*Lactococcus*	MR Egger	0.7944	1.16 (0.39–3.43)	1.0000	
		Weighted median	0.0002	1.56 (1.17–2.08)	0.0269	
		Inverse variance weighted	0.0002	1.44 (1.14–1.83)	0.0137	
GrimAge	*Prevotella7*	MR Egger	0.7160	0.82 (0.29–2.33)	1.0000	0.53
		Weighted median	0.0588	0.80 (0.64–1.01)	0.9998	
		Inverse variance weighted	0.0432	0.84 (0.71–0.99)	1.0000	
GrimAge	*Ruminococcaceae-UCG-010*	MR Egger	0.5421	1.72 (0.35–8.50)	1.0000	
		Weighted median	0.0640	1.69 (0.97–2.93)	0.9522	
		Inverse variance weighted	0.0486	1.54 (1.00–2.37)	0.8259	
GrimAge	*Victivallis*	MR Egger	0.3352	1.91 (0.55–6.59)	1.0000	
		Weighted median	0.1126	1.19 (0.96–1.47)	1.0000	
		Inverse variance weighted	0.0456	1.19 (1.00–1.40)	0.9049	
HannumAge	*Haemophilus*	MR Egger	0.0756	1.83 (1.04–3.23)	1.0000	0.29
		Weighted median	0.0052	1.59 (1.15–2.20)	0.6187	
		Inverse variance weighted	0.0004	1.43 (1.12–1.83)	0.0462	
HannumAge	*Ruminococcaceae-UCG-004*	MR Egger	0.5565	0.60 (0.12–3.04)	1.0000	0.43
		Weighted median	0.1456	0.76 (0.52–1.10)	1.0000	
		Inverse variance weighted	0.0456	0.76 (0.57–0.99)	1.0000	
HannumAge	*Sellimonas*	MR Egger	0.6098	1.31 (0.49–3.51)	1.0000	0.92
		Weighted median	0.1749	1.18 (0.93–1.49)	1.0000	
		Inverse variance weighted	0.0478	1.19 (1.00–1.41)	1.0000	
HannumAge	*Senegalimassilia*	MR Egger	0.2463	2.28 (0.74–7.05)	1.0000	
		Weighted median	0.1435	1.36 (0.90–2.06)	1.0000	
		Inverse variance weighted	0.0211	1.47 (1.06–2.05)	1.0000	
IEEA	*Coprococcus1*	MR Egger	0.2771	1.63 (0.71–3.76)	0.9992	0.72
		Weighted median	0.0346	1.60 (1.03–2.46)	1.0000	
		Inverse variance weighted	0.0320	1.42 (1.03–1.95)	0.7625	
IEEA	*Howardella*	MR Egger	0.2814	1.64 (0.71–3.79)	0.9848	
		Weighted median	0.0313	1.33 (1.03–1.73)	1.0000	
		Inverse variance weighted	0.0240	1.23 (1.03–1.48)	0.9526	
IEEA	*Peptococcus*	MR Egger	0.6249	1.23 (0.55–2.76)	0.9785	0.04
		Weighted median	0.0315	1.35 (1.03–1.78)	1.0000	
		Inverse variance weighted	0.0320	1.24 (1.02–1.52)	0.6356	
IEEA	*Subdoligranulum*	MR Egger	0.0452	3.08 (1.19–7.94)	1.0000	0.10
		Weighted median	0.1246	1.48 (0.90–2.43)	1.0000	
		Inverse variance weighted	0.0253	1.56 (1.06–2.29)	0.7518	
IEEA	*Veillonella*	MR Egger	0.5832	2.81 (0.09–88.38)	0.9774	0.72
		Weighted median	0.1737	1.41 (0.86–2.33)	1.0000	
		Inverse variance weighted	0.0172	1.61 (1.09–2.39)	1.0000	
PhenoAge	*Ruminococcustorques_group*	MR Egger	0.4172	0.56 (0.15–2.09)	0.9547	
		Weighted median	0.0346	0.49 (0.26–0.95)	0.8229	
		Inverse variance weighted	0.0249	0.58 (0.36–0.93)	0.7419	
PhenoAge	*Dorea*	MR Egger	0.2490	2.27 (0.62–8.29)	1.0000	
		Weighted median	0.1570	1.58 (0.84–3.00)	1.0000	
		Inverse variance weighted	0.0279	1.69 (1.06–2.69)	0.6647	
PhenoAge	*Lachnospiraceae-UCG-001*	MR Egger	0.2736	2.32 (0.55–9.72)	1.0000	
		Weighted median	0.0641	1.46 (0.98–2.18)	1.0000	
		Inverse variance weighted	0.0370	1.41 (1.02–1.93)	0.6286	
PhenoAge	*Lachnospiraceae-UCG-008*	MR Egger	0.1223	3.62 (0.83–15.84)	1.0000	
		Weighted median	0.0326	1.54 (1.04–2.30)	0.9684	
		Inverse variance weighted	0.0004	1.51 (1.14–2.00)	0.0512	
PhenoAge	*Lactobacillus*	MR Egger	0.0259	0.27 (0.11–0.69)	1.0000	
		Weighted median	0.0715	0.69 (0.47–1.03)	1.0000	
		Inverse variance weighted	0.0379	0.71 (0.51–0.98)	0.5643	
PhenoAge	*Tyzzerella3*	MR Egger	0.4171	1.71 (0.49–5.95)	0.9732	
		Weighted median	0.0571	1.36 (0.99–1.88)	1.0000	
		Inverse variance weighted	0.0005	1.39 (1.10–1.75)	0.0299	

**Table 2:** Sensitivity analysis of the results of Mendelian randomization of gut microbiota and epigenetic clock.

Outcome	Exposure	MR PRESSO Global_test P value	Cochran’s Q P value	Egger-intercept P value	Steiger
GrimAge	*Ruminococcusgnavus_group*	0.78	0.75	0.32	2.48842E-41
GrimAge	*Dorea*	0.614	0.59	0.29	9.20712E-26
GrimAge	*Eisenbergiella*	0.672	0.64	0.88	3.432E-37
GrimAge	*Lactococcus*	0.22	0.17	0.70	1.79718E-31
GrimAge	*Prevotella7*	0.537	0.50	0.96	7.74447E-42
GrimAge	*Ruminococcaceae-UCG-010*	0.384	0.36	0.89	3.73886E-16
GrimAge	*Victivallis*	0.863	0.85	0.47	3.51908E-40
HannumAge	*Haemophilus*	0.822	0.81	0.38	9.94033E-36
HannumAge	*Ruminococcaceae-UCG-004*	0.503	0.48	0.79	6.47868E-29
HannumAge	*Sellimonas*	0.741	0.74	0.85	5.16753E-37
HannumAge	*Senegalimassilia*	0.899	0.89	0.48	1.2944E-16
IEEA	*Coprococcus1*	0.524	0.46	0.73	8.15555E-34
IEEA	*Howardella*	0.478	0.44	0.52	5.58563E-38
IEEA	*Peptococcus*	0.519	0.47	0.98	5.95127E-43
IEEA	*Subdoligranulum*	0.303	0.27	0.16	1.93328E-29
IEEA	*Veillonella*	0.281	0.23	0.76	3.23535E-18
PhenoAge	*Ruminococcustorques_group*	0.533	0.48	0.96	6.06165E-28
PhenoAge	*Dorea*	0.6	0.58	0.64	3.04292E-26
PhenoAge	*Lachnospiraceae-UCG-001*	0.936	0.92	0.50	4.76372E-40
PhenoAge	*Lachnospiraceae-UCG-008*	0.668	0.64	0.27	2.09483E-37
PhenoAge	*Lactobacillus*	0.363	0.31	0.07	2.37984E-33
PhenoAge	*Tyzzerella3*	0.648	0.62	0.75	4.61924E-48

### The results of gut microbiota and GrimAge

GrimAge has a significant causality with *Ruminococcusgnavus_group* (P = 0.002, Odds Ratio(OR)= 0.78, 95% Confidence Interval(CI) = 0.64–0.97, q = 0.065, rg_p_LDSC_ = 0.043), *Lactococcus* (P = 0.0002, OR = 1.44, 95%CI = 1.14–1.83, q = 0.014).

GrimAge shows a suggestive causality with *Dorea* (P = 0.012, OR = 1.6, 95%CI = 1.11–2.32, q = 0.463), *Eisenbergiella* (P = 0.029, OR = 1.26, 95%CI = 1.02–1.56, q = 0.851, rg_p_LDSC_ = 0.278), *Prevotella7* (P = 0.043, OR = 0.84, 95%CI = 0.71–0.99, q = 1, rg_p_LDSC_ = 0.526), *Ruminococcaceae-UCG-010* (P = 0.049, OR = 1.54, 95%CI = 1.00–2.37, q = 0.826), and *Victivallis* (P = 0.046, OR = 1.19, 95%CI = 1.00–1.40, q = 0.905).

### The results of gut microbiota and HannumAge

HannumAge had a significant causality with *Haemophilus* (P = 0.0004, OR = 1.43, 95%CI = 1.12–1.83, q = 0.046, rg_p_LDSC_ = 0.29).

HannumAge had a suggestive causality with *Ruminococcaceae-UCG-004* (P = 0.046, OR = 0.76, 95%CI = 0.57–0.99, q = 1, rg_p_LDSC_ = 0.435), *Sellimonas* (P = 0.048, OR = 1.19, 95%CI = 1.00–1.41, q = 1, rg_p_LDSC_ = 0.917), *Senegalimassilia* (P = 0.021, OR = 1.47, 95%CI = 1.06–2.05, q = 1).

### The results of gut microbiota and IEAA

IEEA had a suggestive causality with *Coprococcus1* (P = 0.032, OR = 1.42, 95%CI = 1.03–1.95, q = 0.762, rg_p_LDSC_ = 0.72), *Howardella* (P = 0.024, OR = 1.54, 95%CI = 1.00–2.37, q = 0.953), *Peptococcus* (P = 0.03, OR = 1.24, 95%CI = 1.02–1.52, q = 0.636, rg_p_LDSC_ = 0.041), *Subdoligranulum* (P = 0.025, OR = 1.56, 95%CI = 1.06–2.29, q = 0.752, rg_p_LDSC_ = 0.099), *Veillonella* (P = 0.017, OR = 1.61, 95%CI = 1.09–2.39, q = 1, rg_p_LDSC_ = 0.718).

### The results of gut microbiota and PhenoAge

PhenoAge had a significant causality with *Lachnospiraceae-UCG-008* (P = 0.0004, OR = 1.51, 95%CI = 1.14–2.00, q = 0.051), *Tyzzerella3* (P = 0.0005, OR = 1.39, 95%CI = 1.10–1.75, q = 0.03).

PhenoAge had a suggestive causality with *Ruminococcustorques_group* (P = 0.025, OR = 0.58, 95%CI = 0.36–0.93, q = 0.742), *Dorea* (P = 0.028, OR = 1.69, 95%CI = 1.06–2.69, q = 0.665), *Lachnospiraceae-UCG-001* (P = 0.037, OR = 1.41, 95%CI = 1.02–1.93, q = 0.629), *Lactobacillus* (P = 0.038, OR = 0.71, 95%CI = 0.51- 0.98, q = 0.564).

### Sensitivity analysis

IVW, MR-Egger, and weighted median methods show the same causal estimates of direction. There are no outliers in the MR-PRESSO method, and the MR Egger intercept test (P < 0.05) indicates that horizontal pleiotropy does not exist in MR research. Cochran’s Q test (P < 0.05) found no heterogeneity among instrumental variables. Steiger test (P < 0.05) indicated that the direction of MR Analysis was correct and there was no reverse causality.

### Mediation MR Analysis

We used formulas to calculate the direct and mediated effects of inflammatory factors between the gut microbiota and the epigenetic clock (Mediating effect =Beta(XZ) x Beta(ZY); Direct effect = Beta (XY) - Beta (XZ) x Beta (ZY). Among the 91 inflammatory factors, our study found that 4 inflammatory factors met the screening criteria, so mediation analysis was included and the mediation effect and mediation ratio of inflammatory factors were calculated. Beta-nerve growth factor plays a mediating role in *Howardella* and IEAA (mediator effect: -4.08%, direct effect: 25.1%). Oncostatin-M plays a mediating role in *Ruminococcaceae-UCG-010* and GrimAge (mediator effect: -7.71%, direct effect: 59.92%). Interleukin-12 subunit B plays a mediating role in *Prevotella7* and GrimAge (mediator effect: -0.43%, direct effect: -16.96%). C-C motif chemokine 25 plays a mediating role in *Lachnospiraceae-UCG-008* and PhenoAge (mediator effect: -0.35%, direct effect: 41.5%).

## Discussion

In recent years, population aging has posed a global challenge, resulting in increased burdens on national healthcare systems, so we need to explore how to slow down aging and extend life (40). By MR Analysis of four kinds of epigenetic clocks with aging characteristics, genetic correlation with gut microbiota was found. In addition, further mediated MR Analysis identified the inflammatory cytokine pathways that contribute to aging in the gut microbiota. Gut microbiota is associated with aging, providing potential targets for new interventions to promote healthy aging (41). The results of LDSC regression analysis showed that there were suggestive genetic correlations between some epigenetic clock and gut microbiota.

### Potential causal link between epigenetic clock and gut microbiota

Studies have shown that the periodicity and activity of epigenetic clock genes are significantly associated with changes in age (42). Biological aging may be related to the richness and diversity of gut microbiota (12, 13, 43). The results of previous studies are consistent with our MR Analysis in which we found that multiple gut bacteria genera have genetic associations with epigenetic clocks. Higher biological age and lower physical fitness were significantly associated with increased *Dorea* abundance (44). Observational study results have shown a significant increase in *Salmonella* and *Haemophilus* in older individuals (45, 46). *Coprococcus 1* and *Ruminococcus* were found to have the strongest association with age-related phenotypes (47). The relative abundance of *Peptococcus* increased with age (48). *Subdoligranulum* is positively associated with lipopolysaccharide (LPS) biosynthesis and short-chain fatty acid (SCFA) degradation pathways that accelerate epigenetic clock aging (49). An MR Analysis revealed a genetic link between *Veillonella* and longevity (50). At the same time, studies have found that *Lactobacillus* can reduce age-related diseases and regulate the imbalance of gut microbiota (51). The results of MR are different from those of previous studies, which show that the use of *Lactococcus*, and *Lachnospiraceae* can delay aging (52, 53). Due to the few literatures and the influence of confounding factors, this result still needs to be discussed. Interestingly, we also found gut microbiota associated with aging that had not been previously reported, including *Eisenbergiella, Prevotella7, Victivallis, Howardella, Senegalimassilia, and Tyzzerella*. The discovery of these gut microbiota can provide thinking for future scientific research work.

The reduced diversity and abundance of the gut microbiota may be the main reason for the effect of the gut microbiota on the epigenetic clock. It has been found in the literature that the diversity of gut microbiota and the abundance of butyricogenes decreased in the elderly (54–56). The lower bacterial diversity in the elderly showed that Bacteroidetes and Firmicutes still dominated, but the relative proportion of Firmicutes subgroups changed (57). Reducing the pH value of the gut through propionate and butyrate can effectively prevent the overgrowth of pathogens such as *Escherichia coli*, stimulate the growth of beneficial bacteria, and play a regulatory role in the intestinal microbiome (58). However, in the intestinal microbial environment of the elderly, the number of several butyrate-producing gut microbiota is relatively small (such as *Ruminococcus*, etc.). This may lead to the reproduction of intestinal pathogens and the inhibition of beneficial bacteria in the intestine, becoming an important reason for the acceleration of the epigenetic clock.

### Inflammatory cytokines act as mediators of gut microbiota and epigenetic clock

The study found that specific epigenetic features in the DNA of gut microbes in human feces, particularly those associated with inflammation, are strongly associated with disease (59). In our study, we found some possible inflammatory cytokine pathways in the gut microbiota associated with the epigenetic clock. The gastrointestinal tract (GI) and central nervous system (CNS) are constantly confronted with complex human environments. As a result, a complex network of cells, including immune cells and neuronal cells, are able to coordinate local and systemic inflammatory responses (60). Nerve Growth Factor (NGF) modulates the survival, proliferation, and differentiation of neuronal cells within both the peripheral and central nervous systems (61). Some studies have shown that gut microbes can influence levels of NGF in the brain, which in turn affects neurodevelopment and cognitive function (62, 63). Recent studies have shown that the gut-brain axis is able to regulate inflammation and immune responses, thereby influencing the aging process (60, 64). We found that nerve growth factor plays a potential mediating role between gut microbiota and epigenetic clock, and thus may advance the study of the role of gut-brain axis theory in aging. Nerve growth factors regulated by gut microbiota may have potential benefits against neurodegenerative diseases during aging, as these factors are able to protect neurons and slow cognitive decline (65). As a member of the interleukin-6 cytokine family, Oncostatin M (OSM) plays a significant role in inflammation, autoimmune and cancer (66). Specific gut microbes may prompt host cells to restrain Oncostatin-M, which in turn affects inflammatory pathways and immune regulation, mechanisms that may be associated with the aging process, influencing the epigenetic clock by regulating the inflammatory response (67). Interleukin-12 (IL-12) is indispensable in cellular immunity and is considered an effective drug to enhance the anti-tumor immune response. Gut microbiota can influence IL-12B expression through its metabolites or by activating immune cells in the intestinal mucosa. Newly discovered evidence suggests that IL-12B is a key cytokine that enables T helper cells (Th1 and Th17) to differentiate and function (68). Most Th17 and Th1 are present in the gastrointestinal tract and play an important homeostasis role, while positive responses to the flora are thought to be related to inflammation and pathogenesis (69). This effect may indirectly affect the aging process and epigenetic clock by affecting inflammatory states. We found that gut microbiota may control the development of cancer through OSM and IL-12, thus slowing down the effects of aging. C-C motif chemokine 25 (CCL25) is a chemokine that is mainly expressed in the small intestine and plays an important role in attracting immune cells such as T cells to the intestine (70). The composition and function of gut microbiota can influence the intestinal immune environment, including CCL25 expression (71). By regulating the activity of immune cells in the gut, the gut microbiota may indirectly influence the levels of immune regulation and inflammation associated with aging, thereby affecting the epigenetic clock. CCL25 is also involved in the expression of liver inflammatory genes (72). Our findings may be able to control liver inflammation by regulating gut microbiota, thereby delaying aging.

The gut microbiota plays a crucial role in the inflammatory process in the human body (12). In older mice, *Lactobacillus* has been shown to enhance the tight junction of the intestinal barrier, reduce the expression of pro-inflammatory cytokines, and inhibit the activation of NF-kB (73). SCFAs are seen as a central point of connection between the host and the gut microbiota (74). SCFAs can reduce the production of inflammatory factors to achieve immune regulation (75). SCFAs can regulate intestinal transport time, play a role in insulin response, and are closely associated with metabolic diseases (76). SCFAs are an important regulator of microglia integrity in the central system, which is particularly important in older adults and may lead to cognitive decline (77). In addition, there is research evidence that compounds from the gut microbiota can activate macrophages through the blood, putting them into a pro-inflammatory state that leads to atherosclerosis. This may lead to the development of cardiovascular disease (78). The diseases listed above are closely related to human aging, which speeds up the epigenetic clock.

Our study has several advantages: The use of MR Analysis excludes other factors and assesses the genetic association between the epigenetic clock and gut microbiota from a genetic perspective. At the same time, we used LDSC to evaluate the causal link, making the results more reliable. We also used the Steiger test to prove the correctness of the directionality of our study. In addition, in the MR Analysis, we use the F-number to guarantee the strength of the IVs. The MR-PRESSO and MR-Egger regression intercepts can test the horizontal pleiotropy of the study to avoid result bias. European populations were used for exposure and results, avoiding population stratification of results. We used a two-step mediation to determine the role of relevant inflammatory cytokines between gut microbiota and the epigenetic clock.

However, there are limitations to the study. Genus is the lowest classification level in the gut microbiota data, so we were unable to further explore the relationship between exposure and outcome at the species level. Due to the need for the number of SNPs in the sensitivity analysis and horizontal pleiotropy test of this study, our investigation did not achieve the conventional GWAS significance threshold, which is typically set at P < 5 × 10^-8. So we use FDR correction to limit the possibility of positive errors. We only investigated the effect of inflammatory factors as mediators on the epigenetic clock, in fact, the mediators that affect the epigenetic clock may be diverse, such as BMI. Due to the interference of demographic stratification, we analyzed GWAS data from European populations, so the findings may not be applicable to other ethnic groups or populations (79).

## Conclusion

In summary, this two-sample MR Study found a causal relationship between the gut microbiota and the epigenetic clock. Further experimental studies are needed to elucidate the mechanisms by which gut microbiota contribute to the epigenetic clock.

## Data availability statement

The original contributions presented in the study are included in the article/**Supplementary Material**. Further inquiries can be directed to the corresponding author.

## Author contributions

ST: Writing – original draft, Writing – review & editing, Methodology, Formal analysis, Visualization, Resources, Conceptualization. XL: Methodology, Writing – review & editing, Visualization. SC: Methodology, Writing – review & editing. YW: Data curation, Writing – review & editing, Visualization. MC: Data curation, Writing – review & editing, Investigation.
